# Investigation of the activity of 4-aminoquinolines as cysteine protease inhibitors with application in the treatment of Chagas disease

**DOI:** 10.1590/0074-02760240161

**Published:** 2025-02-07

**Authors:** Rahamah Sheu-Idrees, Gabriel Vitor de Lima Marques, Pedro Augusto Lemos Santana, Lucas Abreu Diniz, Daniela de Melo Resende, Saidi Odoma, Omodamiro Olorunshola, Rafaela Salgado Ferreira, Silvane Maria Fonseca Murta, Vinícius Gonçalves Maltarollo, Renata Barbosa de Oliveira

**Affiliations:** 1 Universidade Federal de Minas Gerais, Faculdade de Farmácia, Departamento de Produtos Farmacêuticos, Belo Horizonte, MG, Brasil; 2 Kampala International University, School of Pharmacy, Western Campus, Kampala, Uganda; 3 Kampala International University, School of Pharmacy, Dar Es Salaam, Tanzania; 4 Universidade Federal de Minas Gerais, Instituto de Ciências Biológicas, Departamento de Bioquímica e Imunologia, Belo Horizonte, MG, Brasil; 5 Fundação Oswaldo Cruz-Fiocruz, Instituto René Rachou, Grupo de Genômica Funcional de Parasitos, Belo Horizonte, MG, Brasil; 6 Kogi State University, College of Health Sciences, Department of Pharmacology, Anyigba, Nigeria

**Keywords:** Chagas’ disease, cruzain, TbrCATL, 4-aminoquinoline

## Abstract

**BACKGROUND:**

Chagas disease (CD) is a neglected tropical disease caused by *Trypanosoma cruzi*. The current drugs used to treat these diseases have limited efficacy and produce severe side effects. 4-aminoquinoline derivatives were shown to be a promising class of inhibitors of cysteine proteases cruzain and TbrCATL.

**OBJECTIVES:**

To evaluate the trypanocidal activity of a new series of aminoquinolines as potential inhibitors of cruzain and TbrCATL.

**METHODS:**

Three aminoquinolines were synthesised and their *in vitro* activity was evaluated against cruzain and TbrCATL as well as against amastigotes and trypomastigotes forms of *T. cruzi*. *In silico* studies were also carried out to try to understand the experimental results.

**FINDINGS:**

Compound **5** showed promising activity against cruzain and TbrCATL, with better performance than E60, the reference drug. Compound **5** inhibited cruzain and TbrCATL at IC_50_ of 23 µM ±3 and 29 µM ±1, respectively, but this inhibition showed characteristics of promiscuous inhibition by colloidal aggregation. On the other hand, the compound **4** showed to be more promising activity against *T. cruzi* with IC_50_ 2.57 µM ± 0.03 lower than the reference drug benznidazole 3.8 µM.

**MAIN CONCLUSIONS:**

The results of this study can guide new drug development for the treatment of trypanosomiasis.

*Trypanosoma cruzi* is a flagellate protozoan that causes Chagas disease (CD), also called American trypanosomiasis. *T. cruzi* is mainly transmitted to human by the infected faeces of blood-sucking triatomine bugs, the so called kissing bugs.^1^

CD affects around 6~7 million people, predominantly in Latin America and its incidence is increasing in nonendemic countries due to raised migration.^2^ CD has two clinical phases. The short acute phase is mainly oligosymptomatic with flulike symptoms and the chronic phase persists for the host’s lifespan. In this phase most patients remain asymptomatic, characterising the indeterminate form of CD. Approximately 30-40% develop clinical symptoms with different levels of cardiac and/or digestive tract pathologies (cardiac and digestive forms of CD).^3^

CD is still among the neglected diseases of significant public health issue more than 100 years after it was first discovered.^4^ ^,^ ^5^ This disease frequently strikes those at the height of their productivity, having profoundly negative economic and societal consequences.^1^ ^,^ ^6^ ^,^ ^7^

Currently, benznidazole and nifurtimox are the only drugs available for the clinical treatment of CD. These drugs are recommended in both the acute and chronic phases of CD. However, they have low cure rates mainly during the chronic phase, in addition both drugs present side effects that may result in the interruption of the treatment.^8^ Due to its toxicity, nifurtimox is no longer used in several nations, including Brazil.^9^ ^,^ ^10^

Cruzipain, the main cysteine protease of *T. cruzi*, is expressed in all life-cycle stages of the parasite and plays crucial roles in host cell invasion,^11^ replication,^12^ and modulation of macrophage response.^13^ The term ‘cruzain’ specifically refers to the recombinant form of cruzipain 1, which is part of a multigenic family comprising four cruzipain subtypes.^14^ Several classes of cruzain inhibitors have been described, including vinyl sulfones,^15^ ^,^ ^16^ benzimidazoles,^17^ ^,^ ^18^ aminoquinolines,^19^ ^,^ ^20^ thiosemicarbazones,^21^ ^,^ ^22^ nitrile-based derivatives,^23^ ^,^ ^24^ carbamoyl imidazoles,^25^ and quinazolines.^26^ *TbrCatL*, a homologues protease from *T. brucei*, the causative agent of sleeping sickness or Human African Trypanosomiasis (HAT), is essential for the parasite penetration through the blood-brain barrier and is a validated drug target. Similar to cruzain, several *TbrCat*L inhibitors have been reported, such as bromoisoxazolines,^27^ nitriles,^28^ ^,^ ^29^ thiazoles,^30^ thiosemicarbazones,^31^ trialozes,^32^ vinyl sulfones,^1^ vinyl esters,^33^ and vinyl ketones.^34^ Due to the high similarity between these proteases, evaluating compounds against both can be a promising strategy, potentially leading to the development of different classes of inhibitors that target both proteases.^1^ ^,^ ^19^ ^,^ ^20^ ^-^ ^23^ ^,^ ^35^ ^-^ ^38^ In this study, we aimed to synthesise alternative inhibitors of cruzain and *TbrCatL* that may be reliable, efficient, and viable alternative drugs for treating CD and HAT.

In previous research, Ferreira et al.^39^ used high-throughput screening to discover 146 non-covalent competitive cruzain inhibitors from a compound library of over 190,000 small compounds obtained from the NIH Chemical Genomics Centre (NCGC). One of the most effective compounds was the indole-pyrimidine **1** (Ki = 2.0 µM; IC_50_ = 2.5 µM, Fig. 1), which was then used as a hit compound for the design of new inhibitors from the molecular simplification strategy.^19^ Considering the three fused rings of **1**, analogues were proposed to determine which ring could be removed or replaced without affecting activity. Variations of the side chain length, basicity and substituents were also explored, resulting in the identification of the aminoquinolines **2** and **3** (Fig. 1) as the most promising, obtained in just one synthesis step. Besides, the quinoline derivatives **2** and **3** did not inhibit mammalian cathepsins S and B and were predicted to have good pharmacokinetic and drug-like properties.^19^ ^,^ ^20^

**Fig. 1:**
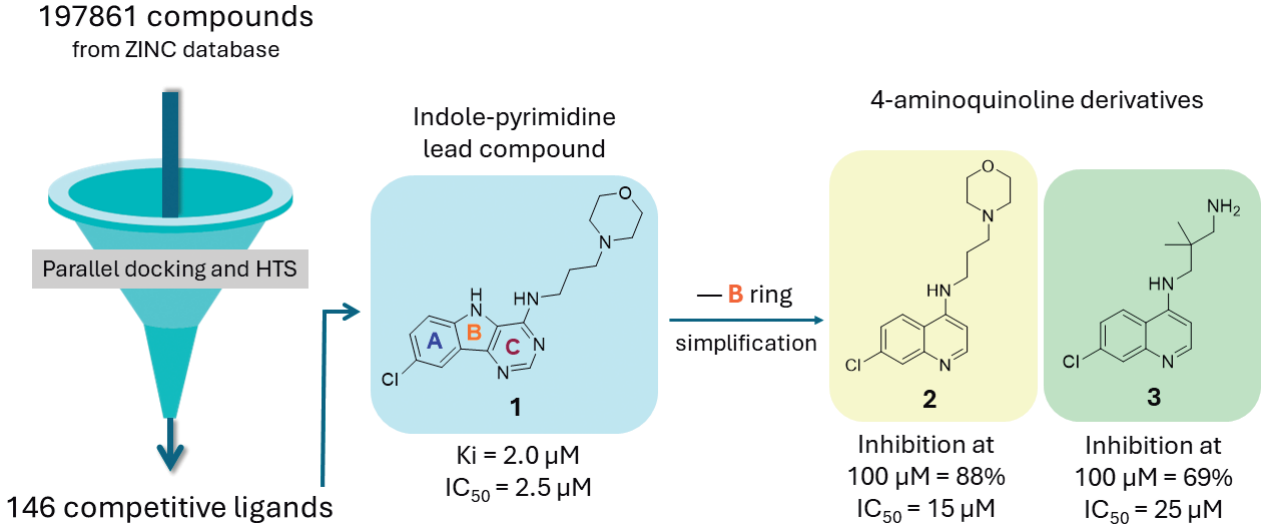
comparison between the structures and activity of indole-pyrimidine **1** and 4-aminoquinolines **2** and **3** identified as cruzain inhibitors.

Continuing these studies, in the present study new 4-aminoquinolines were synthesised by varying the side chain, with the aim of searching for more potent analogues, presenting a greater number of interactions with the molecular target. For the purpose of comparison, three substituents were evaluated in the side chain: a) two hydrophobic substituents containing a cyclic (cyclohexyl) or linear (*n*-butyl) aliphatic chain capable of establishing Van der Waals interactions with the molecular target and b) a heterocyclic substituent containing a heteroatom capable of acting as a hydrogen bond acceptor (furfuryl). It was hypothesised that the cyclohexane ring, being bulkier, would have a better topological fit in a possible hydrophobic pocket, aiding the improvement of binding affinity. On the other hand, the furan ring could reach a possible polar residue. Molecular docking studies were also carried out to investigate the interaction mode of the molecules in the cruzain active site and to find evidence for our hypothesis.

## MATERIALS AND METHODS

*Chemistry* - The synthesis were carried out at Laboratório de Química Farmacêutica, Faculdade de Farmácia, UFMG. Starting materials, reagents and solvents were purchased from commercial suppliers and were used without further purification. The melting point temperatures were determined using a Microquímica MQAPF 301 device. The thin-layer chromatography (TLC) 60 G Merck silica gel was used in 0.25 mm thick layers on glass plates, and iodine vapour was used as a developer to monitor the reaction. NMR Bruker AVANCE III Onebay/Nanobay 400 MHz was used to confirm the structure of the compounds . Chemical shifts are expressed in *δ* (ppm) scale and *J* values are given in Hz, being the multiplicity of signals referred to as singlet (s), doublet (d), doublet of doublets (dd), triplet (t), quartet (q), quintet (qt), and multiplet (m).

Synthesis of quinoline derivatives 4-6

*Synthesis of 7-chloro-N-cyclohexylquinolin-4-amine 4* - To a 50 mL round-bottom flask, connected to a reflux condenser, 100 mg (0.5 mmol) 4,7-dichloroquinoline, 300 mg, (3.1 mmol) of cyclohexylamine and 2 mL of DMF were added. The reaction was maintained under heating at 120ºC and magnetic stirring for 24 h. The reaction progress was monitored by TLC (eluent: ethyl acetate/hexane 7:3; stain: iodine vapour, ninhydrin). After evidence of consume of the starting material, the reaction was cooled to room temperature, and crushed ice added to the round-bottom flask. The formed precipitate was filtered under vacuum. The residue was then washed with 50 mL NaHCO_3_ 0.1 M solution. The filtered residue was stored in a desiccator for drying. The residue was then washed with petroleum ether until the TLC accused a pure compound, obtaining 95 mg (72% yield) of 4 as a brown powder. MP.: 220ºC; NMR data (mixture of ionized and non-ionized form) ^1^H NMR (400 MHz, DMSO-*d6*) *δ* 14.64 (s, 1H), 9.14 (d, 1H, *J* = 7.2), 8.84 (d, 1H, *J* = 9.0), 8.48 (broad s, 1H, N-H), 8.25-8.13 (m, 3H), 7.7 (d, 1H, *J* = 8.6), 6.93 (d, 1H, *J* = 6.5), 1.91-1.06 (m, 22H); ^13^C NMR (100 MHz, DMSO-*d6*) *δ* 154.24, 142.45, 138.65, 137.75, 126.41, 126.27, 125.92, 118.78, 115.31, 98.66, 52.63, 49.19, 31.15, 30.21, 24.89, 24.55, 24.46, 23.69.

*Synthesis of 7-chloro-N-(furan-2-ylmethyl)quinolin-4-amine* **5** - Compound **5** was synthesised according to a reported procedure^40^ and obtained as a dark brown solid (12% yield). M.P.: 195ºC; ^1^H NMR (400 MHz, DMSO-*d6*) ***δ*** /ppm: 8.51 (broad s, 1H, NH), 8.44-8.39 (m, 2H), 7.87 (d, 1H, *J* = 2.2), 7.60-7.59 (m, 1H), 7.52 (dd, 1H, *J* = 9.0 and *J* = 2.2), 6.68 (d, 1H, *J* = 7.3), 6.43-6.39 (m, 2H), 4.59 (d, 1H, *J* = 4.3); ^13^C NMR (100 MHz, DMSO-*d6*) ***δ*** /ppm: 151.17, 151.09, 149.53, 146.39, 142.49, 134.55, 125.39, 124.94, 124.53, 116.99, 110.46, 107.91, 99.22, 39.49.

*Synthesis of N-butyl-7-chloroquinolin-4-amine 6* - Compound 6 was synthesised according to a reported procedure^41^ and obtained as a white solid (67% yield. M.P.: 265ºC, ^1^H NMR (400 MHz, DMSO-*d6*) *δ*/ppm: 8.40 (d, 1H, *J* = 5.6), 8.29 (d, 1H, *J* = 8.8), 7.79 (s, 1H), 7.44 (d, 1H, *J* = 8.8), 7.28 (broad s, 1H, NH), 6.45 (d, 1H, *J* = 5.6), 3.26 (q, 2H, *J* = 7.2), 6.66 (qt, 2H, *J* = 7.2), 1.43 (t, 3H, *J* = 7.2); ^13^C NMR (100 MHz, DMSO-*d6*) *δ*/ppm: 151.85, 149.77, 141.27, 135.35, 128.65, 125.76, 122.01, 117.42, 98.52, 42.08, 29.88, 19.80, 13.73.

*Computational studies* - To better understand how substances might interact with the binding site of cruzain, molecular docking studies were conducted. For this purpose, experiments were performed using the GOLD program version 5.8.1,^42^ and the crystallographic structure of cruzain in complex with a ketoester derivative named as [1-(1-METHYL-4,5-DIOXO-PENT-2-ENYLCARBAMOYL)-2-PHENYL-ETHYL]-CARBAMIC ACID BENZYL ESTER retrieved from the Protein DataBank under the ID 1U9Q.^43^ This structure has acceptable resolution, no sequential mutations or important unmodeled sequences. The binding site was defined as a radius of 10 Å from the centre of mass of the co-crystallised ligand and all crystallographic water molecules were excluded. The simulation protocol, utilising parameters such as the GoldScore scoring function and a number of 200 runs, was validated by the redocking technique.

The software PyMOL (1.99c)^44^ was used for image generation and visual interpretation of the potential interactions. The three-dimensional structures of the compounds were constructed using the Discovery Studio program,^45^ and their most probable ionization states were adjusted to physiological pH using the “fixpka” command of the QUACPAC 1.7.0.2 program.^46^ The lowest energy conformation for each compound was calculated using the OMEGA 2.5.1.4 program.^47^ ^,^ ^48^ The compounds were subjected to simulation following the selection of the optimal experimental protocol, and the highest-ranked poses for each compound were visually inspected.

*Assays against cruzain and TbrCATL* - Recombinant cruzain and TbrCatL were generously provided by Allison Doak and Prof Brian Shoichet (University of California San Francisco, San Francisco, CA, USA) and Prof Conor Caffrey (University of California San Diego, San Diego, CA, USA), respectively. Enzyme activity was measured by monitoring the cleavage of the fluorogenic substrate Z-Phe-Arg-amidomethylcoumarin (Z-FR-AMC) at 25ºC. Fluorescence was monitored at 340/440 nm (excitation/emission) in a Biotec 87 Synergy 2 fluorimeter. Assays were conducted in 96-well flat bottom black plates in 0.1 M sodium acetate buffer, pH 5.5, 10 mM β-mercaptoethanol, 0.01% Triton X-100, 2 nm enzyme and 2.5 µM substrate. DMSO and 1 µM E-64 were employed as negative and positive controls, respectively, in all assays. Assays were performed in triplicate, and enzyme activity was measured for 5 min. Reported values correspond to the mean and standard error of the mean (SEM).

Compounds were initially screened at a concentration of 100 µM against cruzain with and without a 10 min pre-incubation with the enzyme. Screening against TbrCatL was performed at the same concentration with pre-incubation, and only the most potent inhibitor was evaluated in both assay conditions. IC_50_ values were determined from two independent experiments, each with seven compounds concentrations in triplicate. Dose-response curves were built by non-linear regression with Graph Pad Prism 6.

To evaluate the effects of Triton X-100 concentration on enzyme inhibition, assays were conducted using 0.1%, 0.01% and 0% Triton X-100. To access the impact of pre-incubation of bovine serum albumin (BSA) on enzyme inhibition, compounds were incubated with BSA (4 mg/mL, for a final assay concentration of 1 mg/mL) for 10’ followed by 10’ incubation with 2 nM enzyme. Finally, Z-FR-AMC was added to each well and the fluorescence was immediately read.

*Anti-T. cruzi activity assay (amastigotes and trypomastigotes)* - The *in vitro* anti-*T. cruzi* activity was evaluated on L929 cells (mouse fibroblasts) infected with Tulahuen strain of the parasite expressing the *Escherichia coli* β-galactosidase as reporter gene according to the method described previously.^49^ Briefly, for the bioassay, 4,000 L929 cells were added to each well of a 96-well microtiter plate. After an overnight incubation, 40,000 trypomastigotes were added to the cells and incubated for 2 h. Then the medium containing extracellular parasites was replaced with 200 μL of fresh medium and the plate was incubated for an additional 48 h to establish the infection. For IC_50_ determination, the cells were exposed to each synthesized compound at serial decreasing dilutions and the plate was incubated for 96 h. After this period, 50 μL of 500 μM chlorophenol red beta-D-galactopyranoside (CPRG) in 0.5% Nonidet P40 was added to each well, and the plate was incubated for 16 to 20 h, after which the absorbance at 570 nm was measured. Controls with uninfected cells, untreated infected cells, infected cells treated with benznidazole at 3.8 μM (positive control) or DMSO 1% were used. The results were expressed as the percentage of *T. cruzi* growth inhibition in compound tested cells as compared to the infected cells and untreated cells. The IC_50_ values were calculated by linear interpolation. Quadruplicates were run in the same plate, and the experiments were repeated at least once.

*In vitro cytotoxic activity test of compounds and CC*_*50*_ *determination over L929 cell line* - For this bioassay, 4,000 L929 cells in 200 μL of RPMI-1640 medium (pH 7.2-7.4) (Gibco BRL) plus 10% foetal bovine serum and 2 mM glutamine were added to each well of a 96-well microtiter plate that was incubated for three days at 37ºC. The medium was then replaced, and the cells were exposed to compounds at increasing concentrations starting at IC_50_ value for *T. cruzi*. After 96 h of incubation with the compounds the alamarBlueTM was added and the absorbance at 570 and 600 nm was measured after 4-6 h. Controls with untreated and DMSO 1%-treated cells were run in parallel. The results were expressed as the percent difference in the reduction between treated and untreated cells. The compound concentration that inhibits 50% of the L929 cell viability (CC_50_) was determined. Quadruplicates were run in the same plate and the experiments were repeated at least once.

IC_50_ over *T. cruzi* and L929 cells were determined by linear interpolation and the selectivity index (SI) was calculated by the ratio of CC_50_ L929 cells/ IC_50_ *T. cruzi*.

## RESULTS AND DISCUSSION

**Fig. 2:**
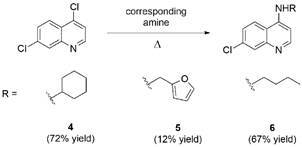
synthetic route for the synthesis of 4-aminoquinlines.

The 4-aminoquinolines **4**, **5** and **6** were synthesised by nucleophilic replacement of 4,7-dichloroquinoline with cyclohexylamine, furfurylamine and *n*-butylamine, respectively (Fig. 2). Compounds were experimentally evaluated against cruzain and TbrCATL.

Initial screening was performed at 100 μM with and without a 10-minute pre-incubation with the enzyme. The observed percentages of inhibition were similar under both conditions, indicating that the inhibition is not time-dependent (Table I). Among the evaluated molecules, compound **5** was the most potent, exhibiting 99% inhibition against cruzain and 94% against TbrCatL.

**TABLE I:** Cruzain and TbrCATL inhibition at 100 µM of each compound

Compound	% Inhibition at 100 µM (Mean ± SEM)			
	Cruzain		TbrCATL	
	10’ pre-incubation	No pre-incubation	10’ pre-incubation	No pre-incubation
4	18 ± 2	19 ± 1	38 ± 4	-
5	99 ± 1	93 ± 1	94 ± 1	87 ± 3
6	35 ± 1	35 ± 1	42 ± 4	-

SEM: standard error of the mean.

**Fig. 3:**
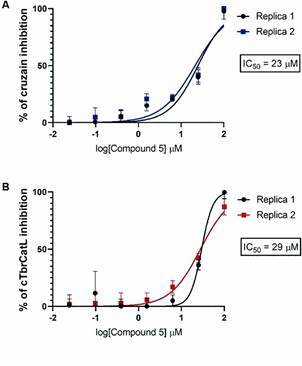
dose-response curves for the inhibition of cruzain (A) and TbrCatL (B) by compound 5. Each IC_50_ curve was built based on assays at seven compound concentrations, in triplicates. Two independent experiments were performed for IC_50_ determination.

We determined the IC_50_ values of compound **5** against cruzain (IC_50_ = 23 ± 3 μM) and TbrCatL (IC_50_ = 29 ± 1 μM, Fig. 3).

To investigate aggregation as an undesirable mechanism of enzyme inhibition, we employed two well-established experiments. First, the compound was evaluated at two concentrations of Triton X-100 (0.1% and 0.01%) and in the absence of this detergent. Triton X-100 disrupts small molecule aggregates.^50^ ^,^ ^51^ Compound **5** showed sensitivity to the detergent, with reduced inhibition observed at higher detergent concentrations (Table II). Additionally, pre-incubation of an aggregator with bovine serum albumin saturates the protein-binding capacity of the aggregate. We observed reduced inhibition for compound **5** after pre-incubation with BSA, suggesting that the compound forms aggregates (Table II). These results suggest that the inhibition observed might be artefactual, resulting from colloidal aggregation, and not from specific inhibition of the enzymes.

**TABLE II:** Confirmatory assays in varying Triton X-100 concentrations and in the presence or absence of bovine serum albumin (BSA)

Compound	Evaluated concentration (µM)	% Inhibition of cruzain (Mean **±** SEM)			
Triton 0.1%	Triton 0.01%	Triton 0%	BSA +Triton 0%
5	23	28.4 ± 3	28.4 ± 2	100 ± 1	32.2 ± 1

SEM: standard error of the mean.

Additionally, the activity of compounds on the amastigote and trypomastigote forms of *T. cruzi* in cell culture was evaluated using the Tulahuen strain expressing beta-galactosidase. The three compounds tested showed activity, with compound **4** standing out as the most potent, with an IC_50_ value of 2.57 μM (Table III). Unfortunately, this compound demonstrated cytotoxicity towards uninfected L929 cells (CC_50_ < 2.30 μM, SI < 8.9). However, additional modifications to the structure of **4** can be made in an attempt to increase selectivity, making this compound a hit for further studies.

**TABLE III:** *In vitro* effect of compounds on the growth of *Trypanosoma cruzi* and on uninfected fibroblasts

Compound	*T. cruzi* IC_50_ (µM)	L929 cells CC_50_ (µM)	SI^*a*^
4	2.57 ± 0.11	< 2.30	< 8.9
5	143.14	-	-
6	149.92	-	-
Benznidazole^*b*^	3.8 ± 0.8	2,381	626

*a*: SI (selectivity index) is the ratio of murine fibroblast viability (CC_50_) to the IC_50_ on *T. cruzi*; *b*: positive control.

**Fig. 4:**
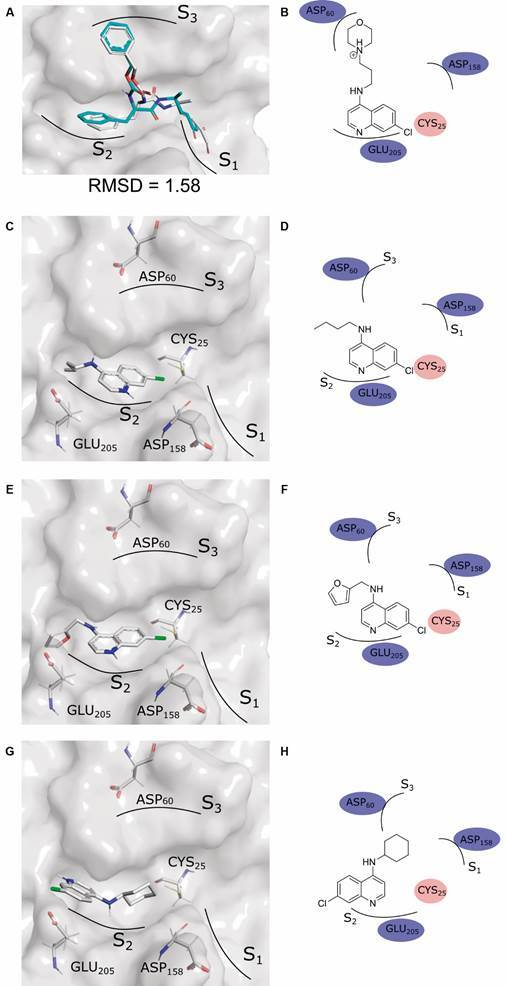
potential interactions between quinolinic derivatives and the cruzain binding site. (A) Pose generated by redocking (PDB-ID 1U9Q); (B) Proposed two-dimensional interactions for analogue 2 by Martins et al.;^53^ (C and D) Potential three- and two-dimensional interactions of substance **4**, respectively; (E and F) Potential three- and two-dimensional interactions of substance **5**, respectively; (G and H) Potential three- and two-dimensional interactions of substance **6**, respectively.

After the experimental evaluation of compound **4-6** biological activities, the compounds’ potential interactions with the cruzain’ active site were proposed using molecular docking. The best protocol was validated by the redocking technique, which generated a pose with an RMSD equals to 1.58 Å (Fig. 4A), indicating an agreement between predicted and experimental binding modes.^52^ The predicted interactions were visually analysed and compared among themselves and with the interactions predicted for the aminoquinoline derivative **2**, reported by Martins et al.^53^ and referred to here as the positive control (Fig. 4B).

It can be seen that the quinoline group of the control (Fig. 4B) and the tested derivatives (Fig. 4C-H) are accommodated in the S2 region of the cruzain binding site. This region is reported as essential for the affinity and selectivity of enzyme inhibitors.^1^ ^,^ ^53^ However, it is observed that other pockets of the interaction site (such as S1 and S3) are not occupied by the tested analogues, but only by the control and co-crystallised ligand. This difference is primarily due to the larger structure of the control (which reaches the mentioned regions) and the ionization of the molecules, since the control is a morpholine derivative, positively charged at physiological pH, which favours the interaction with Asp_60_. Similarly, the cysteine 25 (CYS_25_) residue shows potential interaction with the quinoline groups of the control and compounds **5** and **6**, but not with compound **4**, which, being inverted in the site, precludes such interaction. This difference is likely due to the cyclohexane group, which, being bulkier, would better accommodate in the S2 pocket and, being at the opposite end of the quinoline ring, would favour inversion.

The differences in interactions with the pockets of the interaction site between the proposed analogues and the control (and aminoquinoline derivatives) may explain the differences in affinities observed in experimental tests. The *in-silico* results suggest the possibility of exploring new aminoquinoline derivatives that have groups capable of interacting with the S1 and S3 pockets (using positively ionizable groups, for example).

In conclusion, in this study, aminoquinoline **4** was identified with potent trypanocidal activity and could, in the future, have its structure optimised to increase its SI. The aminoquinolines synthesised in this work were not capable of inhibiting the cysteine proteases cruzain and TbrCATL, thus requiring further investigation of their possible mechanism of action.
